# Systems Biology Understanding of the Effects of Lithium on Cancer

**DOI:** 10.3389/fonc.2019.00296

**Published:** 2019-04-30

**Authors:** Weihao Ge, Eric Jakobsson

**Affiliations:** ^1^National Center for Supercomputing Applications, University of Illinois at Urbana-Champaign, Urbana, IL, United States; ^2^Center for Biophysics and Computational Biology, University of Illinois at Urbana-Champaign, Urbana, IL, United States; ^3^Department of Molecular and Integrative Physiology, University of Illinois at Urbana-Champaign, Urbana, IL, United States

**Keywords:** lithium, systems biology, biochemical pathways, biochemical networks, cancer, gsk3b, kinases, phosphotransferases

## Abstract

Lithium has many widely varying biochemical and phenomenological effects, suggesting that a systems biology approach is required to understand its action. Multiple lines of evidence point to lithium as a significant factor in development of cancer, showing that understanding lithium action is of high importance. In this paper we undertake first steps toward a systems approach by analyzing mutual enrichment between the interactomes of lithium-sensitive enzymes and the pathways associated with cancer. This work integrates information from two important databases, STRING, and KEGG pathways. We find that for the majority of cancer pathways the mutual enrichment is statistically highly significant, reinforcing previous lines of evidence that lithium is an important influence on cancer.

## Introduction

### Clinical and Epidemiological Context for Lithium and Cancer

By far the most common medical use of lithium is as a first line therapy for bipolar disorder, including associated depression as well as mania (1). A comprehensive review of the literature confirms that lithium is also effective against unipolar depression with unique anti-suicidal effectiveness, and may also be useful against cancer and neurodegenerative disease (2, 3).

One line of evidence for the possible use of lithium as an anticancer agent is epidemiological. A retrospective study showed that psychiatric patients undergoing lithium therapy for bipolar disorder had a much lower incidence of cancer than a matched group not receiving lithium therapy (4). More recent studies of similar design, one conducted nationwide across Sweden, and another across Taiwan, achieved the same result (5, 6). On the other hand another nationwide study, this time from Denmark, showed no correlation of lithium with colorectal adenocarcinoma (7). On closer look, the Denmark study does not contradict the Swedish study. The Swedish study also found that for the entire population lithium was not correlated with cancer incidence, but in addition found that bipolar individuals not treated with lithium had a higher incidence of cancer than the general population. Lithium-treated bipolar patients, on the other hand, had essentially the same cancer incidence as the general population.

One piece of experimental evidence for lithium's potential as a cancer therapeutic modality is that it was observed to inhibit prostate tumor growth (8), presumably through its ability to inhibit GSK3. A detailed study of molecular mechanisms by which lithium inhibition of GSK3-beta inhibits proliferation of prostate tumor cells in culture was presented by Sun et al. (9). The work was subsequently extended to an animal model (10). A clinical trial for the effect of lithium coupled with prostatectomy on men has been conducted but as of this writing results have not yet been published ^1^.

With respect to other cancers, lithium has been found to be lethal to neuroblastoma cells but not to normal nerve cells (11). A similar effect was found in ovarian cancer cells (12), although a subsequent similar study on ovarian cancer cells suggests only a more modest benefit (13). It is not clear from our reading of the two ovarian cancer papers why the results are significantly different from each other.

With respect to colorectal cancer, one study suggests that lithium inhibits proliferation of a colorectal cancer cell line (14). Another study on colon cancer cells showed that lithium specifically induced a reversal of the epithelial-to-mesenchymal transition characteristic of the cancer cells (15).

Two studies with relatively small sample size suggested a possible link between lithium and tumors of the upper urinary tract (16, 17). However, a large-scale study involving all urinary tract cancers in Denmark over a multi-year period found no correlation with lithium use (18).

Because lithium therapy is systemic rather than topical or local, it follows that lithium might inhibit metastasis. Evidence that this is the case for colon cancer comes from observation of inhibition of metastasis-inducing factors by lithium and by observation on reduced metastasis in model animals given lithium therapy (19).

Autophagy is a key cellular process in the inhibition of cancer (20). Lithium has been shown to induce autophagy, due to its inhibition of inositol monophosphatase (21). The full range of lithium effects on autophagy is complicated (22), as might be expected because lithium has multiple targets, which themselves have multiple substrates.

A promising strategy is to combine lithium with other therapies. Many cancer therapies have side effects, so augmentation with lithium may have the dual effect of enhancing the effect of the therapy itself and also permit lower but still effective therapeutic doses. Han et al. (23) reported success in combining lithium with another GSK3 inhibitor against TP53 wild-type glioblastoma cells. Zhukova et al. (24) reported that lithium abrogated TP53-mutant radiation resistance in medulloblastomas, showing that effectiveness of radiation therapy may be enhanced by augmenting with lithium.

Because of the promising indications as cited above, lithium has been suggested as one of a number of drugs commonly used for other reasons, to be repurposed for cancer (25).

### Biochemical Context for Lithium and Cancer

Much of lithium's known biochemical action may be summarized by noting that it inhibits some phosphate-transfer enzymes (primarily phosphatases and kinases) that have magnesium as a co-factor (2). A common underlying biophysical basis for competition between lithium and magnesium for modulating phosphate-transfer enzymes, is suggested by noting that the primary energy source for cells and the substrate for phosphorylating enzymes is not bare ATP, but rather magnesium-associated ATP (MgATP) (26). NMR studies show that lithium associates with MgATP (27). Based on this admittedly small amount of data, we consider the possibility that lithium generally associates with magnesium-phosphate complexes and thus has the potential to modulate to some extent a large number of phosphorylation reactions and ATP-splitting processes.

Because mutations in G protein linked receptors have emerged as of interest in cancer research (28), it is significant that lithium appears to inhibit β-adrenergic and muscarinic receptor coupling to G proteins by competing with magnesium, which facilitates such coupling (29–33).

In the literature we find evidence for direct lithium inhibition of 17 human magnesium-dependent phosphate-transfer enzymes, as follows: A review by Phiel and Klein (34) identified five (IMPase, IPPase, FBPase, BPntase, and GSK3B). Testing against a panel of 80 protein kinases (35) revealed lithium sensitivity for eight more enzymes (MNK1, MNK2, smMLCK, PHK, CHK2, HIPK3, IKKϵ, and TBK1). It has long been observed that adenyl cyclase activity is inhibited by lithium (36). Of nine different adenylyl cyclases tested, two (ADCY5 and ADCY7) are strongly inhibited by lithium and one (ADCY2) is less strongly but significantly inhibited (37). With the addition of GSK3A (38), we have a list of 17 phosphate-transfer enzymes directly inhibited by lithium. An inspection of protein-protein interaction databases indicates that all 17 interact with multiple other gene products. It should be noted that 72 out of 80 kinases (33), and six out of nine adenylyl cyclases (34), screened were found *not* to be lithium-sensitive. Table 1 provides the names and synonyms, including both the commonly used gene and protein names, for the 17 known human lithium-sensitive enzymes.

**Table 1:** Names and synonyms for the known human lithium-sensitive enzymes and the genes that code for them, derived from entries in the UniProt database.

**Names and synonyms of human lithium-sensitive enzymes**	**Functional class of enzyme**
GSK3A, Glycogen synthase kinase-3 alpha	Kinase
GSK3B, Glycogen synthase kinase-3 beta	Kinase
MNK1, MKNK1, MAP kinase-interacting serine/threonine-protein kinase 1, MAP kinase signal-integrating kinase 1	Kinase
MNK2, MKNK2, MAP kinase-interacting serine/threonine-protein kinase 2, MAP kinase signal-integrating kinase 2, MAPK signal-integrating kinase 2, GPRK7	Kinase
smMLCK, Myosin light chain kinase-smooth muscle, MYLK, Telokin, MLCK	Kinase
PHK, Phosphorylase b kinase gamma catalytic chain, PHK-gamma, PHKG2, PSK-C3, Phosphorylase kinase subunit gamma-2	Kinase
CHK2, Serine/threonine-protein kinase Chk2, CHEK2, CHK2 checkpoint homolog, Hucds1, hCds1, CDS1, RAD53	Kinase
HIPK3, Homeodomain-interacting protein kinase 3, Androgen receptor-interacting nuclear protein kinase, ANPK, Fas-interacting serine/threonine-protein kinase, FIST, DYRK6, FIST3, PKY	Kinase
IKKϵ, Inhibitor of nuclear factor kappa-B kinase subunit epsilon, IKBKE, I-kappa-B kinase epsilon, IKK-E, IKK-epsilon, IkBKE, Inducible I kappa-B kinase, IKK-I, IKKE, IKKI, KIAA0151	Kinase
TBK1, Serine/threonine-protein kinase TBK1, NF-kappa-B-activating kinase, T2K, TANK-binding kinase 1, NAK	Kinase
IMPase, Inositol monophosphatase 3, IMPAD1, IMP 3, IMPase 3, Golgi 3-prime phosphoadenosine 5-prime phosphate 3-prime phosphatase, Golgi-resident PAP phosphatase, gPAPP, Inositol monophosphatase domain-containing protein 1, Inositol-1(or 4)-monophosphatase 3, Myo-inositol monophosphatase A3	Phosphatase
IPPase, Inositol polyphosphate 1-phosphatase, IPP, INPP1	Phosphatase
FBPase, Fructose-1,6-bisphosphatase 1, FBPase 1, D-fructose-1,6-bisphosphate 1-phosphohydrolase 1, Liver FBPase, FBP, FBP1	Phosphatase
BPntase, BPNT1, 3′(2′),5′-bisphosphate nucleotidase 1, Bisphosphate 3′-nucleotidase 1, PAP-inositol 1,4-phosphatase, PIP	Phosphatase
ADCY2, Adenylate cyclase type 2, ATP pyrophosphate-lyase 2, Adenylate cyclase type II, Adenylyl cyclase 2, KIAA1060	Adenyl cyclase
ADCY5, Adenylate cyclase type 5, ATP pyrophosphate-lyase 5, Adenylate cyclase type V, Adenylate cyclase type V, AC5	Adenyl cyclase
ADCY7, Adenylate cyclase type 7, ATP pyrophosphate-lyase 7, ATP pyrophosphate-lyase 7, Adenylate cyclase type VII, Adenylyl cyclase 7, KIAA0037	Adenyl cyclase

Because lithium affects many different biological molecules and processes (2), it is essential to utilize the tools of systems biology (39) if a comprehensive understanding of lithium action and its prospects for therapy are to be obtained. Important concepts for organizing biological information in a systems context are pathways and networks. A very useful tool for obtaining data about known pathways is the KEGG database (40). An equally useful and complementary tool is the STRING database of interacting proteins (41).

In the present paper we investigate further the possible linkages between lithium and cancer by analyzing the mutual enrichment between STRING-derived interactomes of lithium-sensitive enzymes, and the KEGG pathways associated with cancer.

## Methods

Analysis was performed on the interactomes of the above-mentioned lithium-sensitive proteins. The interactomes of these proteins were extracted from the STRING database of protein-protein interactions (https://string-db.org). For each key protein, we adjust confidence level and order of neighbors (nearest only or next nearest included), so that each set contains a few hundred proteins. This size is large enough for statistically reliable enrichment analysis.

### Disease Association

We used the R-package KEGGgraph (42, 43) to identify the proteins and genes associated with the cancer-relevant pathways.

### *P*-Value Calculation

The fundamental question we address is whether there is significant overlap or mutual enrichment between the interactomes of lithium-sensitive proteins and the pathways implicated in various cancers. Both the interactomes and the pathways are represented by lists of their constituent gene products (proteins). Our methods and results should be considered in the context of the publication from the American Statistical Association, “The ASA's Statement on *p*-values: Context, Process, and Purpose” (44) In particular the statement notes “*P*-values and related analyses should not be reported selectively. Conducting multiple analyses of the data and reporting only those with certain *p*-values (typically those passing a significance threshold) renders the reported *p*-values essentially uninterpretable.” We acknowledge that such selective reporting is common—indeed the ASA statement was prompted in large measure by the widespread practice of such selective reporting. In this paper we will present in graphical form **all** the *p*-values we compute, so that their significance can be judged in complete context.

**Figure 1:**
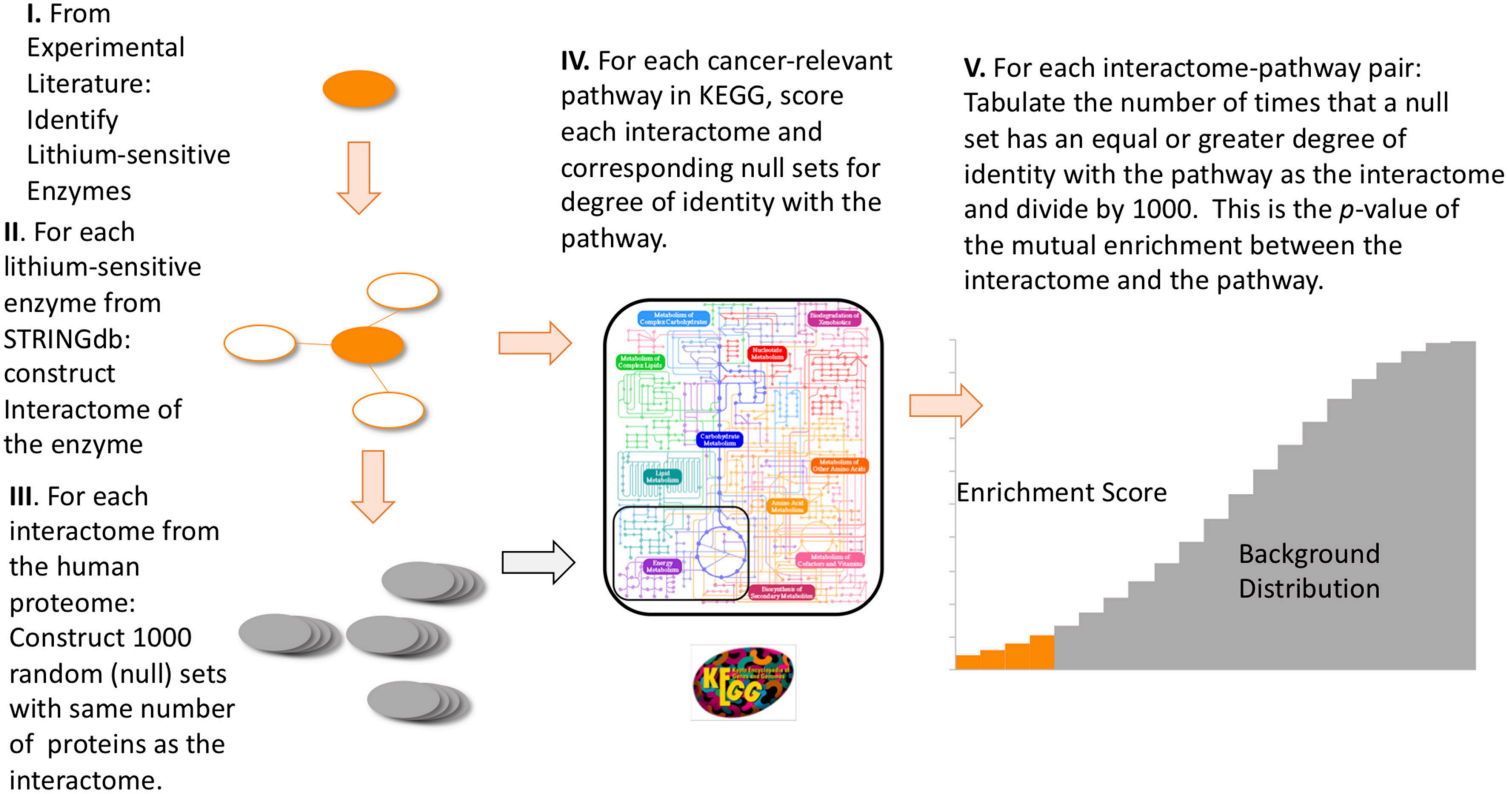
Flow diagram of the steps to compute empirical *p*-values for the mutual enrichment between the interactomes of lithium-sensitive enzymes and cancer-relevant pathways. The steps are: (1) From the experimental literature, identify the lithium-sensitive enzymes, (2) using the STRING database, construct the interactome of each of the lithium-sensitive enzymes, (3) For each interactome, construct 1,000 null sets consisting of proteins randomly chosen from the entire human proteome with each null set containing the same number as the interactome, (4) for each interactome and null set, calculate the degree of identity with the list of proteins from each cancer-relevant pathway, (5) for each interactome-pathway combination, tabulate the number of times the null set has equal or greater degree of identity with the pathway set than does the interactome. This number, divided by 1,000, is the *p*-value for mutual enrichment between the interactome and the pathway.

For each of the 17 lithium sets, an ensemble of 1,000 null sets are generated by random selection from the human genome. Each null set is the same size as the corresponding lithium set. Then we used the R-package STRINGdb (45) to perform KEGG pathway enrichment analysis. This operation is a particular example of the powerful technique of gene-annotation enrichment analysis (46). In gene-annotation enrichment analysis a test list of genes (often derived from gene expression experiments) or proteins is compared to an organized database of gene annotations, often referred to as a gene ontology (47), an array of gene lists corresponding to different biological functions, molecular functions, or locations in the cell. Although the phrase “gene ontology” is used to describe the database, in fact the objects in the database are the gene products, or proteins, whose biological function, molecular function, and location in cell are tagged with annotations. The output of the gene/protein-annotation enrichment analysis is expressed as the likelihood that the list overlaps could have occurred by chance (*p*-value). A very low *p*-value implies that the degree of overlap is highly significant statistically and very likely is significant biologically. In our study the lists we are comparing are the proteins contained in the interactomes of lithium sensitive enzymes on the one hand, and the proteins and genes contained in the KEGG pathways associated with cancer on the other hand. For each KEGG term retrieved, a null distribution of uncorrected *p*-value is generated by the 1,000 null sets. This gives us a measure of the false discovery rate, since any overlap between the null sets and the KEGG pathways is purely accidental. Then the fraction of null set uncorrected *p*-values smaller than or equal to the lithium-sensitive set uncorrected *p-*value would be the empirical *p*-value. A flow chart for this calculation is provided in Figure 1. For a detailed discussion of empirical *p*-value determination see Ge et al. (48).

## Results

**Figure 2:**
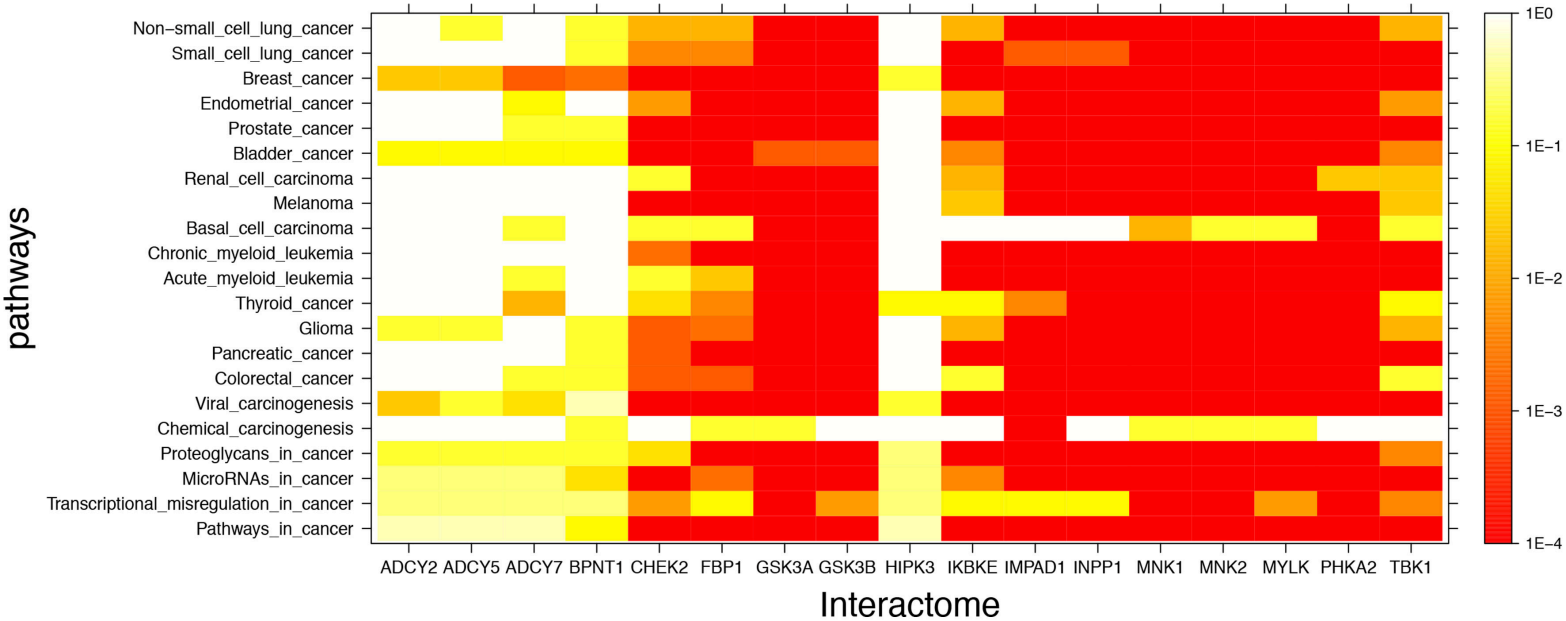
Visual representation of mutual enrichment patterns between specific cancer pathways and the interactomes of lithium-sensitive gene products. Calibration of *p*-value vs. color is indicated by a vertical scale to the right of the heat map. Red or dark orange indicates very strong enrichment while lighter color indicates weak or, if white, no enrichment. Five genes stand out as being not strongly connected to these cancer pathways: BPNT1, HIPK3, ADCY2, ADCY5, and ADCY7. Of the cancer pathways, chemical carcinogenesis stands out as being less likely to be strongly influenced by lithium levels, although there is a strong mutual enrichment between the interactome of IMPAD1 and this pathway. For the remainder of the genes and the remainder of the cancers, the relationship between the lithium-sensitive interactome and the cancer phenotype is strong.

Figure 2 shows mutual lithium interactome enrichment with specific cancer pathways, represented by heatmaps. Each area on the heatmap is a color-coded representation of the degree of mutual enrichment between the genes in the interactome of the indicated lithium sensitive enzyme and the genes in the indicated pathway. The darker the shade, the more significant the mutual enrichment of the interactome-pathway combination is. The light areas on the heatmap represent situations where a lithium-sensitive interactome has little or no mutual enrichment with a cancer pathway. The dark areas, deep orange and red, represent situations where enrichment is very strong—far greater than could be expected by chance and therefore statistically highly significant. The deep red color between 1E-3 and 1E-4 on the vertical scale is uniform and represents the situation where not even one of the 1,000 null sets was as mutually enriched with the pathway as was the interactome.

It appears that the interactomes of five out of the 17 lithium-sensitive genes (ADCY2, ADCY5, ADCY7, BPNT1, and HIPK3) do not show significant mutual enrichment with the cancer pathways explored in this study. Chemical carcinogenesis shows significant mutual enrichment with only one of the interactomes, that of IMPAD1. For the remaining specific cancer pathways and lithium-sensitive interactomes, there are multiple areas of strong mutual enrichment, as indicated by deep orange to red coloring. The genes contained in these overlapping areas, and their modes of regulation, appear worthy of further study in unraveling the details of the lithium vs. cancer relationship.

**Figure 3:**
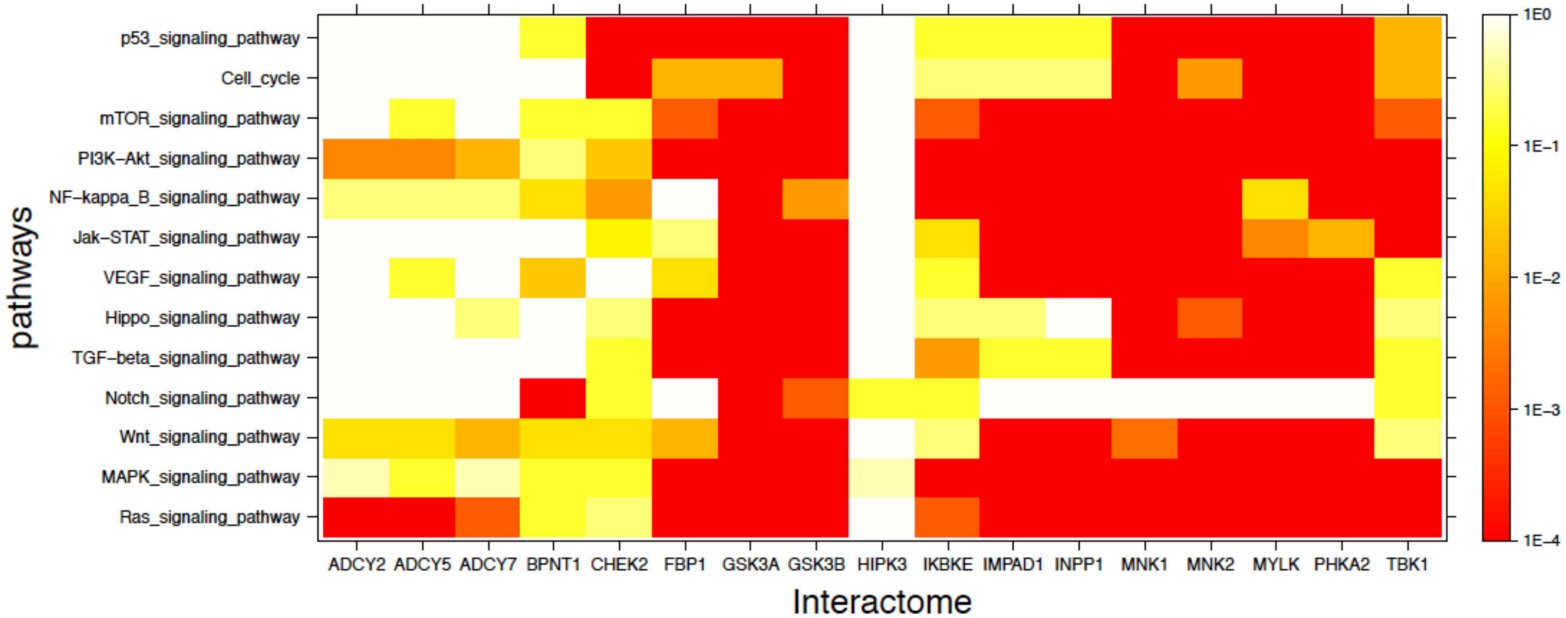
Visual representation of mutual enrichment patterns between signaling pathways implicated in cancer and the interactomes of lithium-sensitive gene products. Calibration of *p*-value vs. color is indicated by a vertical scale to the right of the heat map. Red or dark orange indicates very strong enrichment while lighter color indicates weak or, if white, no enrichment. Only one gene product appears not relevant to cancer, HIPK3. The three adenyl cyclases, BPNT1, and CHEK2 show strong mutual enrichment for a couple of the pathways. Each of the remaining 11 interactomes show strong mutual enrichment with most of the cancer-relevant pathways.

In addition to the labeled specific cancer pathways we extended the analysis to signaling pathways in which dysfunction is implicated in cancer, as indicated in the literature (49–56). Figure 3 shows in heatmap form the mutual enrichment between the 17 lithium-sensitive interactomes and 13 pathways relevant to cancer. In Figure 3 we find that the interactomes of the lithium sensitive enzymes ADCY2, ADCY5, ADCY7, and BPNT1 that did not show strong enrichment in any of the pathways for specific cancers do in fact show strong enrichment with some cancer-relevant signaling pathways. Specifically, ADCY2, ADCY5, and ADCY7 show strong enrichment with the Ras signaling pathway and BPNT1 shows strong enrichment with the Notch signaling pathway. Only HIPK3 remains without strong enrichment with any relevant pathway.

The inescapable conclusion from Figures 2, 3 is that variability in lithium concentration is likely to significantly modulate most cancer-relevant pathways. We should note that sensitivity to lithium does not necessarily imply a *beneficial* sensitivity. There are some indications for some cancers that lithium might be beneficial, as described in the Introduction section of this paper, but because of the complexity of the feedback relationships in these pathways, a complicated relationship between lithium ingestion and cancer incidence is very possible.

## Summary and Discussion

We have conducted a pathway and network enrichment analysis exploring the role of lithium in multiple cancers and cancer-related pathways. The results show that for the large majority of such cancers, there is high mutual enrichment between the interactomes of lithium-sensitive enzymes and the pathways associated with those diseases, indicating that lithium is very likely to affect the incidence and course of the disease. Our results are consistent with a variety of lines of evidence from both epidemiology and from experiment, cited in earlier sections of this paper, suggesting possible influence of lithium on the incidence and progression of cancer.

We hope that the results described in this paper will contribute to prioritizing and designing clinical trials of lithium for cancer. To provide context for such prioritization and design, it is essential to take into account the ways in which lithium is unique, both as a pharmaceutical and as an ion that is ubiquitous in the environment, and therefore ubiquitous in the water and food we ingest (2):

1. Unlike other ions, lithium is not closely regulated by selective membrane transport processes. Rather it shares transport and permeation pathways that are mainly selective for other ions, in most cases sodium (2). Therefore, lithium concentration in both extracellular and intracellular compartments, rather than being nearly constant as is the case with other ions, is roughly proportional to lithium ingestion (57). Whereas, changes in the concentrations of other ions of more than a few percent have severe acute adverse consequences, the human body adjusts without acute adverse consequence to changes in lithium concentrations of several orders of magnitude. Our biochemistry has evolved to accommodate to widely varying lithium levels, as opposed to developing the ability to closely regulate lithium levels.
2. The multiple enzymes inhibited by lithium are each functionally linked to large numbers of other genes. This explains why the effects of lithium are widespread and varied; lithium has a modulating effect on many gene networks. We note that screening for lithium sensitivity has so far not included systematic examination of multiple variants of particular gene products, either mutational variants or alternative splices from the same gene. Therefore, it may be that some of the enzymes that have been found not lithium-sensitive may have mutational or splice variants that are sensitive. Conversely, some of the enzymes that have been found to be lithium-sensitive may have mutational or splice variants that are insensitive. The plausibility of such a possibility is exemplified by a functional, structural, and mutational study on an archaeal inositol monophosphatase (58). The archaeal enzyme has high homology (30% identical, 50% similar) to its human counterpart and functions in the same magnesium-dependent manner. In this study it was shown that a single amino acid substitution could convert the enzyme from its native lithium-insensitive form to a lithium-sensitive form. Perhaps of relevance, it has long been known that lithium responsiveness is significantly variable among human individuals (59).
3. Unlike other pharmaceuticals, lithium is probably an essential trace element in the diet (60–62). The question with lithium is not whether it should be ingested or not, but rather how much. Extreme lithium deprivation results in failure to thrive, while too much lithium is toxic. The existence of these extrema suggests existence of an intermediate optimum.

Therefore, we suggest that the correct question to ask with respect to lithium and a particular disease is not, “Should lithium be administered for this particular disease?” but rather, “What is the optimum blood level of lithium for this individual, given his or her disease history, status, genetic propensities, and other medications?” Unlike some pharmaceuticals that are more specific and inhibit or activate one gene or a small number of genes, the model for lithium action is that it alters the balance between a large number of interacting processes and pathways. Thus, a dose-response curve for lithium is likely to be highly non-linear and not always monotonic.

There are just a few well-established markers for optimum concentrations. For a patient with a reliable diagnosis of bipolar disorder a common target for optimality would be blood concentration of 0.8–1 mM. Significantly higher concentrations will result in acute toxicity, while significantly lower will result in loss of effectiveness. However, this level has some side effects when sustained for years or decades, namely an increased risk of kidney damage and lowered thyroid activity (63).

At the other end of the dosage scale, epidemiological evidence is compelling that geographical variations in concentration of lithium in the drinking water are correlated with a variety of health and wellness markers, most notably and reliably with incidence of suicide (64–69).

Another important marker is provided by a study showing that over a 4-year period a lithium level of 0.25–0.4 mM of lithium (1/4 to 1/2 of the bipolar therapeutic dose) did not incur any renal damage (70). This study suggests that clinical studies exploring low to medium-dose lithium could be undertaken with relatively minimal concerns for side effects.

One possible piece of low-hanging fruit for a clinical trial would be low- to medium-dose lithium for men undergoing active surveillance (AS) for advance of prostate cancer. From studies of AS outcomes, a large fraction of patients on AS ultimately require invasive treatment, as reviewed by Dall'Era et al. (71). When this need arises it typically comes after only a few years. Thus, a trial of lithium in this context would produce significant results in a short time and would be relatively inexpensive.

A second area that seems ready for clinical trial is augmentation of other cancer therapies, either radiation or pharmaceutical, with low-to-moderate lithium. Studies we have cited in this paper support the possibility of beneficial results, and also support the lack of side effects from such lithium doses.

## Author Contributions

The work was planned jointly in conversations between EJ and WG. WG did the computations and prepared the figures and tables. WG wrote the first draft of the Methods and Results sections. EJ wrote the first draft of the Introduction and Conclusions sections. Both authors shared in the final refinement of the manuscript.

### Conflict of Interest Statement

The authors declare that the research was conducted in the absence of any commercial or financial relationships that could be construed as a potential conflict of interest.

## Footnotes

^1^https://clinicaltrials.gov/ct2/show/NCT02198859
